# Role of Exercise in Visceral Adipose Tissue Inflammation and Macrophage Polarization in Hypertensive Mice

**DOI:** 10.3390/ijms27010251

**Published:** 2025-12-25

**Authors:** Venkata Polaki, Harshal Sawant, Brody Pinson, Cindy Zhu, Shuzhen Chen, Ji Chen Bihl

**Affiliations:** 1 Department of Pharmacology and Toxicology, Boonshoft School of Medicine, Wright State University, Dayton, OH 45435, USA; vpolaki@wisc.edu; 2 Department of Biomedical Sciences, Joan C. Edwards School of Medicine, Marshall University, Huntington, WV 25755, USA; sawantha@marshall.edu (H.S.); pinson34@marshall.edu (B.P.); zhu36@marshall.edu (C.Z.); chens@marshall.edu (S.C.)

**Keywords:** hypertension, exercise, adipose tissue, inflammation, mice, macrophages, polarization

## Abstract

Macrophages accumulate in visceral adipose tissue (VAT) during hypertension and may contribute to hypertension-associated inflammation. Exercise has shown beneficial effects on hypertension; however, the exact mechanisms by which the activated immune cells lead to the protective effects remain unclear. Our study aimed to determine how exercise influences VAT inflammation by modulating the macrophage polarization in hypertensive mice. Renin transgenic (R+) mice were used as a hypertensive mouse model and subjected to exercise (8 weeks). The body weight and blood pressure were monitored, VAT morphology was assessed by H&E and Masson Trichrome staining, macrophage polarization was determined by immunostaining and flow cytometry, and macrophage phenotype-related proteins were analyzed within the VAT via Western Blots. Results showed that exercise reduced the adipocyte size and collagen content of VAT and increased cell infiltration in R+ mice. Immunostaining and flow cytometry data showed that the ratio of pro-inflammatory macrophages (M1) to anti-inflammatory macrophages (M2) was increased in the VAT of R+ mice, while exercise corrected the macrophage polarization, which was consistent with protein level changes in VAT. Together, our data suggest that exercise improves vascular remodeling and VAT function (reduced adipocyte size, loss of collagen) by modulating VAT inflammation (polarization of macrophages) in hypertensive mice.

## 1. Introduction

Hypertension is a growing public health problem worldwide. According to the WHO, 1.13 billion people worldwide are hypertensive, and nearly half of adults in the United States (108 million, or 45%) have hypertension. Alarmingly, only about 1 in 5 adults (20%) with hypertension have adequate control of their condition [1]. Global health initiatives have set a target to reduce the prevalence of hypertension by 25% by 2025 (using 2010 as the baseline). Hypertension is a global pandemic; if untreated, it may lead to end-organ damage commonly known as congestive heart failure, myocardial infarction, stroke, and chronic kidney disease. Therefore, novel strategies for prevention are needed.

Adipose tissue (AT) is distributed throughout the body, and is broadly classified into white AT and brown AT, of which 20–25% of the total body weight constitutes white AT. AT was previously known to be a protective layer, but recent studies have shown that AT plays a role as an endocrine organ [2,3,4]. AT is not only an energy storage site but is also known to secrete paracrine, endocrine, and autocrine molecules. Visceral adipose tissue (VAT) is known to be infiltrated with inflammatory cells like macrophages during hypertension. In VAT, lipotoxic effects are seen where the ability to metabolize fats is overwhelmed, which results in the accumulation of immune cells like macrophages [4,5]. An increased composition of VAT is known to be strongly associated with vascular diseases and elevated blood pressure. It releases different molecules like reactive oxygen species, proinflammatory cytokines, and free fatty acids. The release of these molecules results in increased blood pressure by causing vasoconstriction and decreased vasodilation, fluid retention, and vascular remodelling [5].

Exercise is one of the primary non-pharmacological therapies, also known as lifestyle modification therapies, in the treatment of hypertension. Studies have shown that regular physical training can lower blood pressure by approximately 5–7 mmHg [6,7,8]. Beyond its hemodynamic benefits, exercise has been shown to alleviate inflammation in AT in high-fat diet (HFD)-fed mice by inducing a macrophage phenotype switch from M1- to M2-macrophages and preventing macrophage infiltration in the AT [9,10]. In this study, we aimed to investigate whether treadmill exercise intervention could alleviate inflammation of VAT through macrophage polarisation, thereby contributing to improving the vascular injury/function in hypertensive mice.

Macrophages in adipose tissue (ATMs) are crucial for inflammation in VAT, especially concerning metabolic and cardiovascular issues like hypertension. In hypertensive conditions, VAT experiences immune remodeling marked by heightened recruitment of pro-inflammatory macrophages, increased cytokine production, adipocyte enlargement, and extracellular matrix (ECM) accumulation. These alterations lead to diminished adipose function and encourage systemic vascular dysfunction by changing adipokine secretion and increasing oxidative stress. Previous research has indicated that renin-induced hypertension enhances macrophage infiltration into VAT and activates inflammatory signaling pathways that aggravate metabolic and vascular damage [11,12].

Recent studies highlight that ATMs do not align with a rigid M1/M2 polarization model but rather reflect a range of activation states influenced by adipocyte stress, metabolic signals, and microenvironmental factors [13]. Physical activity has become a powerful modulator of immune composition in adipose tissue, with research indicating that exercise affects not only classically activated macrophages but also various resident, infiltrating, and perivascular macrophage subsets in metabolic tissues. Exercise can significantly alter ATM functional programs to favor pro-resolving and tissue-repairing states, reduce inflammatory cytokine production, and enhance adipocyte morphology [14]. Nonetheless, the majority of current research has focused on inflammation related to obesity, and there is limited understanding of how physical activity influences macrophage activation and VAT structure, particularly in the scenario of renin-dependent hypertension.

Consequently, this study tackles an important knowledge gap by examining whether treadmill exercise can reduce VAT inflammation and modify macrophage activation states in a hypertensive mouse model. Through the combination of histological, biochemical, and immune-profiling methods, we examine the impact of exercise on adipocyte size, ECM remodeling, and macrophage phenotype in VAT, as well as whether these tissue-level alterations relate to enhancements in vascular remodeling. This study offers new perspectives on immune modulation from exercise in hypertension, enhancing existing knowledge of ATM diversity and adipose-vascular interactions.

Therefore, this study addresses an important knowledge gap by investigating whether treadmill exercise can reduce VAT inflammation and modify macrophage activation states in a hypertensive mouse model. Through the combination of histological, biochemical, and immune-profiling methods, we examine the impact of exercise on adipocyte size, ECM remodeling, and macrophage phenotype in VAT, as well as whether these changes in tissue correlate with enhancements in vascular remodeling. This study offers new perspectives on immune modulation from exercise in hypertension, enhancing existing knowledge of ATM diversity and adipose–vascular interactions.

## 2. Results

### 2.1. The Effects of Exercise Intervention on Decreasing VAT Weight in Hypertensive Mice

**Figure 1:**
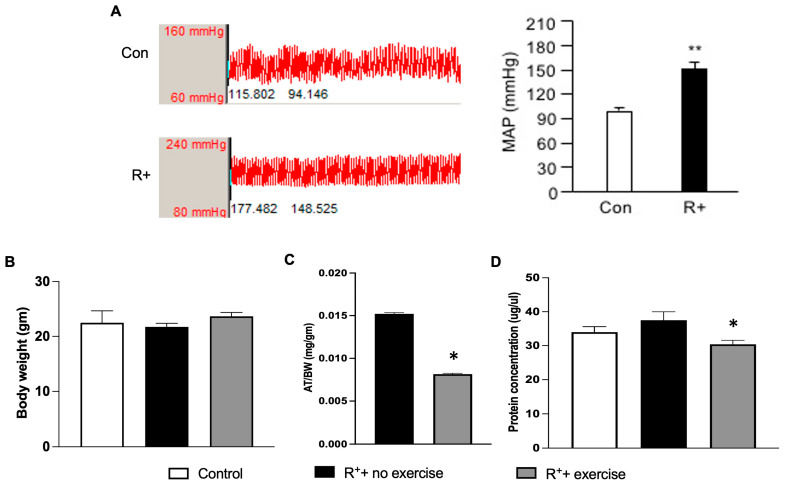
**The effects of exercise on the weight and the protein concentration of VAT in a hypertensive mouse model.** (**A**) Characterization of the hypertensive animal model (R+). The MAP was significantly increased in the R+ mice, indicating that the animals were hypertensive. (**B**) Summarized data depicting body weights of animals in three groups. (**C**) The ratio of VAT weight and BD in different groups. (**D**) The effect of exercise on protein level in the VAT. Data are represented as mean ± SEM. *n* = 11. * *p* < 0.05, ** *p* < 0.01 vs. control (wild type). MAP: mean arterial pressure; AT: adipose tissue; BD: body weight.

First of all, the hypertensive mouse model (R^+^) was confirmed by measuring the MAP. As shown in Figure 1A, the MAP in R^+^ mice was significantly increased when compared to the wild-type control mice (*p* < 0.05). The bodyweight of the mice was recorded before and by the end of each week of exercise. The summarized data in Figure 1B showed that there was no significant difference in body weight between the different groups.

To reveal the relationship between the weight of the VAT to the body weight, we also calculated the ratio of VAT weight to the body weight. As shown in Figure 1C, the ratio of VAT weight to body weight was decreased significantly in the exercised hypertensive group compared to the control group, suggesting that the VAT is changed in exercised hypertensive mice (*p* < 0.05, vs. R^+^ + no exercise). These results suggest that exercise helped in decreasing the VAT growth of hypertensive mice. Moreover, the effects of exercise intervention on VAT protein levels in hypertensive mice were also investigated. As shown in Figure 1D, there was a significant decrease in the level of protein concentration of VAT in the exercise group when compared to the no-exercise group (*p* < 0.05, vs. R^+^ + no exercise). No significant differences between control and R^+^ + no exercise in the protein concentration of VAT were observed.

### 2.2. The Effects of Exercise Intervention on the Structure of the Aorta

**Figure 2:**
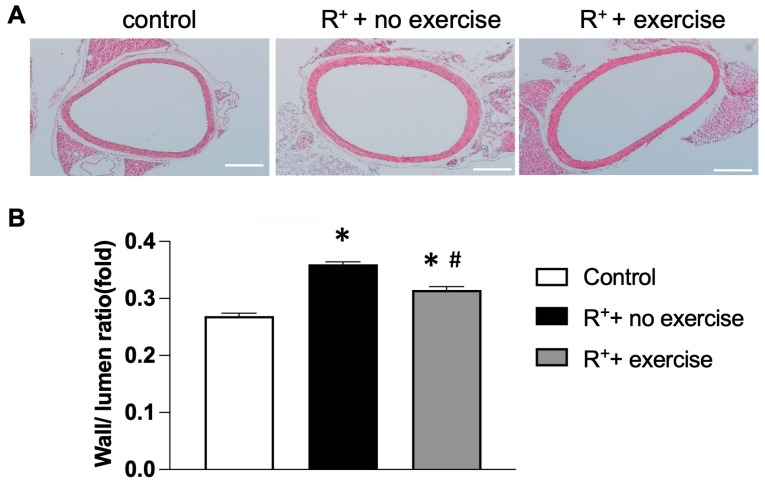
**The effects of exercise on aorta remodelling in a hypertensive mouse model.** (**A**) Representative images of aorta remodelling illustrated by H&E staining. (**B**) Summarized data on the Wall/lumen ratio changes after exercise. Data are represented as mean ± SEM. *n* = 5. * *p* < 0.05 vs. control (wild type), ^#^ *p* < 0.05 vs. R^+^ + no exercise.

During the condition of hypertension, the vessels undergo vascular remodeling. Sections of the aorta were stained with H&E to determine their structure and remodeling (Figure 2A). Images were taken at 10× magnification, and the remodeling (wall-to-lumen ratio) was calculated using ImageJ. As shown in Figure 2B, the wall-to-lumen ratio was higher in hypertensive mice without exercise when compared to the control group (*p* < 0.05, vs. control). After exercise, the wall-to-lumen ratio has been remarkably reduced in the aorta of hypertensive mice (*p* < 0.05, vs. R^+^ + no exercise). Hence, an eight-week treadmill exercise intervention altered the aortic structure in hypertensive mice.

### 2.3. The Effect of Exercise Intervention on VAT Morphology in Hypertensive Mice

**Figure 3:**
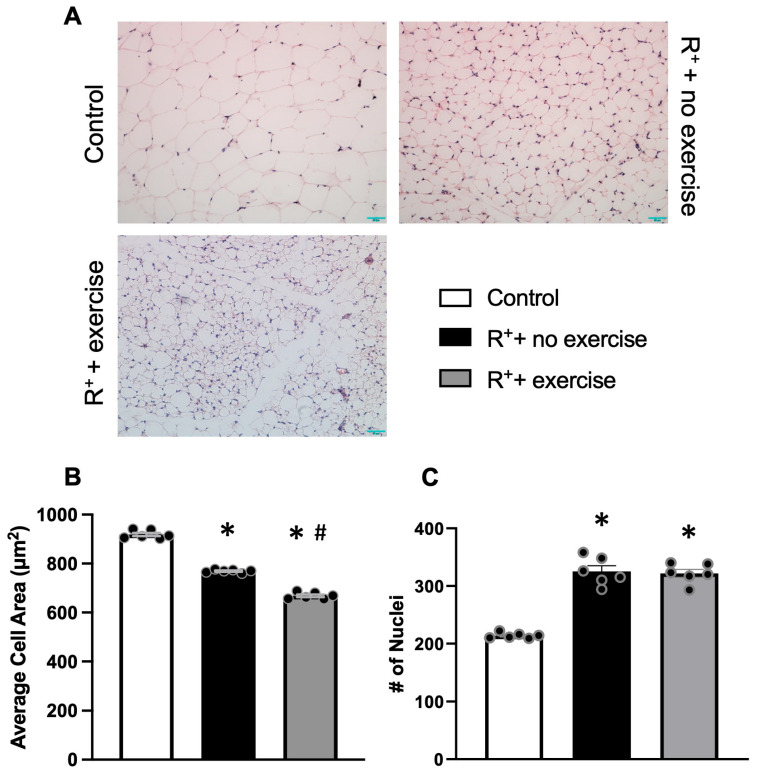
**The effects of exercise on histological changes in VAT in a hypertensive mouse model.** (**A**), Representative images of VAT illustrated by H&E staining. (**B**), The average cell area of individual adipocytes in VAT from different groups. (**C**), The cell nuclei numbers in VAT from different groups. Data are represented as mean ± SEM. *n* = 6. * *p* < 0.05 vs. control (wild type), ^#^ *p* < 0.05 vs. R^+^ + no exercise.

To observe the effect of exercise on the morphology of VAT, we performed H&E staining of VAT. In adipose depots of sedentary control mice, the adipocyte area was significantly enlarged (Figure 3A). The results showed a significant reduction in adipocyte size in the hypertensive mice (*p* < 0.05, vs. control, Figure 3B). Moreover, the exercise training could further reduce the adipocyte area in the hypertensive mice (*p* < 0.05, vs. R^+^ + no exercise, Figure 3B). It also has been observed that the infiltrated cell numbers counted by the number of nuclei per image area were increased in the hypertensive mice (*p* < 0.05, vs. control, Figure 3C) but yielded no statistically significant differences after exercise training (Figure 3C).

### 2.4. The Effect of Exercise Intervention on Collagen Content of VAT in Hypertensive Mice

**Figure 4:**
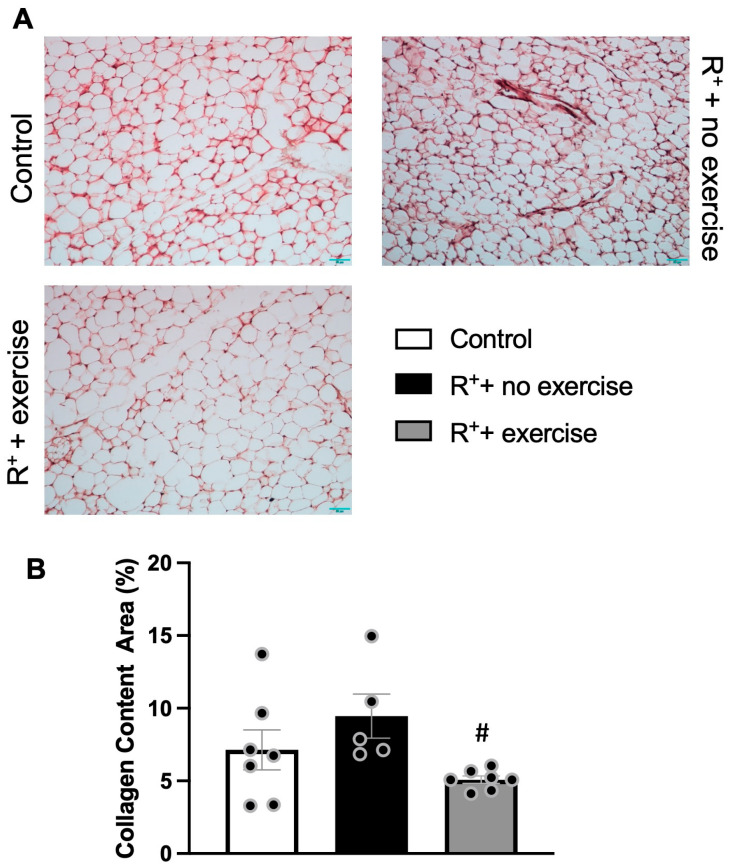
**The effects of exercise on collagen content of VAT in a hypertensive mouse model.** (**A**) Representative images of collagen content in VAT illustrated by Trichrome staining. (**B**) The bar chart showing collagen content in VAT in different groups. Data are represented as mean ± SEM. *n* = 7. ^#^ *p* < 0.05 vs. R^+^ + no exercise.

The Masson Trichrome staining was used to measure the percentage of image area taken up by collagen proteins between neighboring cell membranes in the extracellular matrix (ECM) in VAT. The representative images from each experimental group were included in Figure 4A, and the intensity of the expression level was calculated as shown in Figure 4B. The data showed that there was an increase in collagen content in VAT in the hypertensive mice (*p* < 0.05, vs. control). This might correlate to the pro-inflammatory status in hypertensive mice, leading to fibrosis and impaired VAT. Interestingly, the collagen content was significantly decreased in the hypertensive mice with exercise training (*p* < 0.05, vs. R^+^ + no exercise), suggesting that exercise might provide a beneficial effect on matrix remodeling in VAT during hypertension.

### 2.5. The Effect of Exercise on Macrophage Polarization in VAT

**Figure 5:**
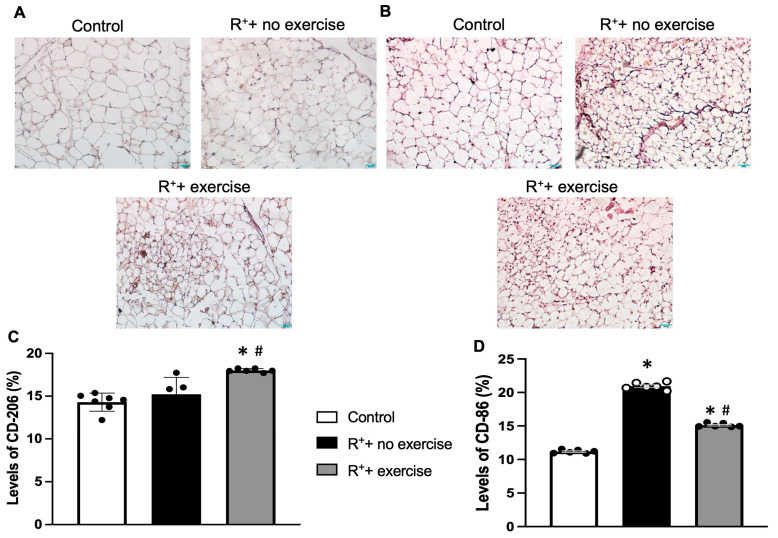
**Effect of exercise on macrophage polarization in VAT.** (**A**,**B**) Representative images of IHC staining with M1/M2-specific markers in VAT. (**C**) The percentage of CD206-positive areas in the VAT. (**D**) The percentage of CD86-positive areas in the VAT. Data are represented as mean ± SEM. *n* = 7. * *p* < 0.05 vs. control (wild type), ^#^ *p* < 0.05 vs. R^+^ + no exercise.

Immunohistochemistry was used to assess macrophage phenotypes in VAT slides, including M1 and M2. For M1 (pro-inflammatory), CD86 was used, and CD206 was used for M2 (anti-inflammatory). The representative images from each experimental group were shown in Figure 5A. The intensity of the expression level was calculated and that data showed that there was no significance observed between the control and hypertensive mice regarding the M2 level. However, the exercise training was able to significantly increase the M2 level in the hypertensive mice (*p* < 0.05, vs. R^+^ + no exercise, Figure 5B). On the other hand, there was an increase in M1 in the hypertensive mice (*p* < 0.05, vs. control, Figure 5C), suggesting the pro-inflammatory status was increased in the VAT in the hypertensive mice. More importantly, the M1 level was significantly decreased in the hypertensive mice with exercise training (*p* < 0.05, vs. R^+^ + no exercise, Figure 5C).

**Figure 6:**
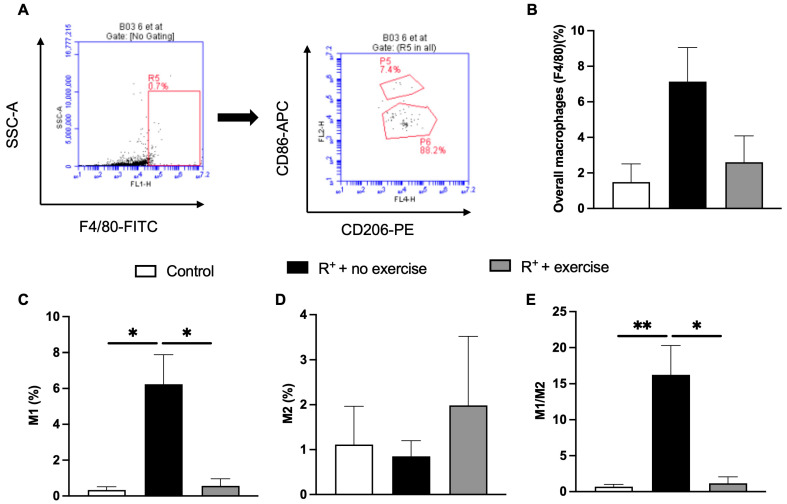
**Effect of exercise on macrophage phenotype changes in VAT.** (**A**) Representative plot of flow cytometry for macrophages (F4/80+ -- FL1-H) in VAT from exercised hypertensive mice. Representative plot of flow cytometry for M1 (CD86+ -- FL2-H) and M2 (CD206+ -- FL4-H) macrophages in VAT from exercised hypertensive mice. (**B**) The population of M1 and M2 macrophages in VAT. The percentage of macrophages (F4/80+). (**C**) The percentage of M1 (CD86+). (**D**) The percentage of M2 phenotype (CD206+). (**E**) The ratio of M1/M2. Data are represented as mean ± SEM. *n* = 5. * *p* < 0.05, ** *p* < 0.01.

Flow cytometry was also used to determine the type of markers and receptors on the surface of the cells by using labelled antibodies. After 8 weeks of exercise, the number of macrophages (F4/80 positive cells) from all three groups of mice in VAT was analyzed. F4/80 positive cells were gated out from all the cells (Figure 6A). The CD86 and CD206 markers were used to identify M1 and M2 phenotypes, respectively. Figure 6B showed that the overall numbers of macrophages were higher in hypertensive mice without exercise when compared to the control group, which decreased after 8 weeks of exercise (no significant difference). Figure 6C showed there was a higher percentage of M1 macrophages (F4/80+CD86+ cells) in the VAT of hypertensive mice than that in control mice (*p* < 0.05, vs. control) and the percentage of M1 was significantly decreased after exercise (*p* < 0.05, vs. R^+^ + no exercise, Figure 6E). However, a higher M2 percentage was detected in the VAT of exercised hypertensive mice as compared to that in the non-exercised (Figure 6D), though no statistical significance was observed.

**Figure 7:**
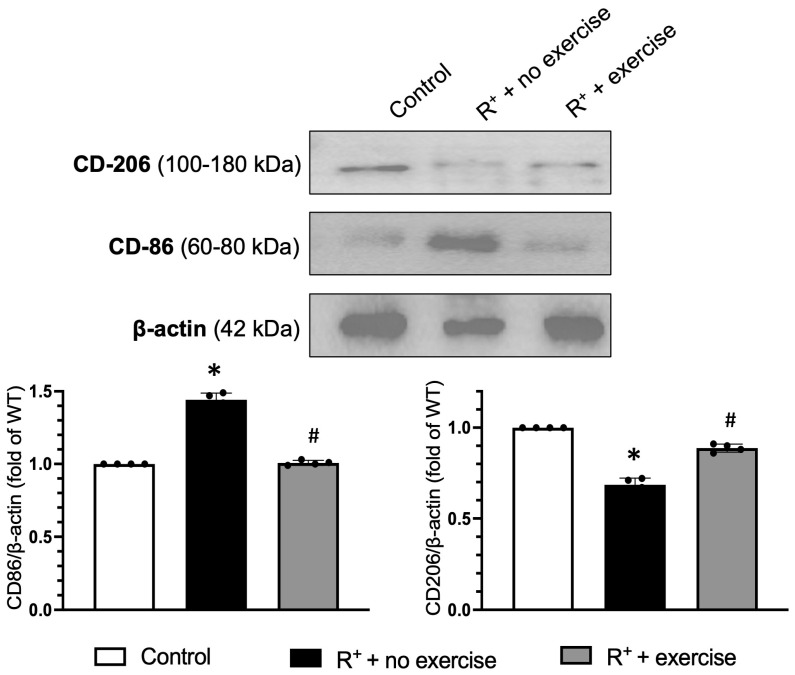
**Effect of exercise on macrophage polarization protein levels in VAT.** The protein expression level of type 1 (M1) macrophage maker (CD86) is increased, while the expression level of type 2 (M2) macrophage maker (CD206) is decreased in the VAT from R+ hypertensive mice. Exercise could reverse these protein levels. Data are represented as mean ± SEM. *n* = 4. * *p* < 0.05 vs. control (wild type); ^#^ *p* < 0.05 vs. R^+^ + no exercise.

Moreover, Western Blot analyses were used to confirm the macrophage phenotype protein expression levels in the VAT in different groups. The data showed that the hypertensive mice expressed significantly elevated levels of CD86 (M1, pro-inflammatory cytokine) as compared to the other groups (*p* < 0.05, vs. control), and the hypertensive exercised groups showed a decrease in CD86 in comparison to the hypertensive group (*p* < 0.05, vs. R^+^ + no exercise, Figure 7), running consistent with the exercise theory of reducing inflammation with increased physical activity. For the anti-inflammatory phenotype, M2 levels had an inverse effect, with the CD206 expression level decreased in the hypertensive mice (*p* < 0.05, vs. control), while exercise could up-regulate the CD206 expression in the VAT (*p* < 0.05, vs. R^+^ + no exercise, Figure 7).

## 3. Discussion

Exercise is widely recognized for its beneficial effects on vascular disease and hypertension [15]. However, the exact mechanisms of how it benefits the vascular system are still incompletely understood. Previous reports have shown that the content and size of the adipocytes are changed after exercise [16,17]. Hence, VAT may play a role in maintaining the overall positive effect of exercise during many cardiovascular disorders. The VAT undergoes inflammation during hypertension with an accumulation of macrophages and many other cytokines and chemokines [18]. After accumulating in the VAT, macrophages undergo differentiation into M1 and M2 phenotypes [19]. The M1 phenotype has pro-inflammatory effects, while the M2 phenotype has anti-inflammatory effects. In this study, we aimed to test whether exercise can attenuate VAT inflammation in hypertension and to investigate whether this effect involves the modulation of macrophage polarization within VAT. Specifically, we used renin transgenic mice (R+), as they have high blood pressure with renin expression at a constant rate in the liver, which leads to elevated levels of renin and prorenin in the plasma. We confirmed the animal model by showing the high MAP in the R+ mice when compared to the controls. These mice were subjected to treadmill exercise intensity (10 m/min for 8 weeks, 5 days a week), which was based on our previous reports showing the beneficial effects on vascular diseases [20,21,22].

To assess vascular pathological changes associated with hypertension, we measured the structure of aorta (and remodeling) in the hypertensive mice. As the vessel undergoes remodeling in hypertension [23], previous studies have shown that exercise induces vascular remodeling by inducing angiogenesis and arteriogenesis [24]. Thus, this was also our interest to see if exercise could provide beneficial effects on vascular remodeling. As expected, the H&E staining results showed pronounced aortic remodeling in hypertensive mice. Furthermore, we found that exercise decreased aorta remodeling in hypertensive mice after exercise. This is the first time we found that exercise could benefit the vascular system by alleviating aorta remodeling in the R+ hypertensive mouse model.

For the physiological effects, we measured the body weights before, during (weekly), and after the exercise. Firstly, we noticed that the body weight of the hypertensive mice is slightly higher than the normal control mice, which agrees with previous studies showing that spontaneous hypertensive rats have lower body mass than normotensive control rats at various ages [25]. Secondly, an increase in the body weights of the exercise group was observed when compared to the control and the no-exercise hypertensive group, while there were no significant differences between the groups. Our data suggest that exercise had no significant effect on the body weights of hypertensive mice. Treadmill exercise studies in spontaneously hypertensive rats (SHRs) show that exercise can lead to a reduction in body weight compared to sedentary groups, particularly in older rats. Some studies observe no significant change in body weight, while others note that exercised SHR may have a lower body weight than their sedentary counterparts or normotensive controls, with the specific outcome depending on the exercise protocol, age, and intensity [25,26,27,28].

Hypertension causes distinct changes in visceral adipose tissue, such as adipocyte enlargement, greater extracellular matrix accumulation, and enhanced activation of pro-inflammatory macrophages [29,30]. These results reinforce the increasing acknowledgment that VAT inflammation directly leads to systemic vascular dysfunction and metabolic issues. Our findings are consistent with earlier studies showing hypertensive VAT remodeling, while also broadening these insights to a renin-dependent model. A major finding of this research is that exercise modifies the immune environment of hypertensive VAT via mechanisms that go beyond the traditional M1/M2 framework. While CD86 and CD206 served as functional markers for classically and alternatively activated macrophage states, our findings should be seen in the larger context that ATMs exist along a spectrum of activation phenotypes. Exercise is recognized for influencing various macrophage subsets such as resident, infiltrating, and perivascular populations instead of solely affecting one polarization axis. Our results showing elevated CD206 expression and decreased CD86-related signaling correspond with growing evidence that physical activity enhances pro-resolving macrophage roles and mitigates VAT inflammation across various metabolic scenarios.

A significant finding of this research is showing that physical activity changes the immune environment of hypertensive VAT via mechanisms that go beyond the traditional M1/M2 model. While CD86 and CD206 function as operational indicators of traditionally activated and alternatively activated macrophage states, these markers only reflect a segment of a wider activation spectrum. Exercise reshapes various macrophage subsets including resident, infiltrating, and perivascular populations rather than affecting just one polarization axis. Our observations of elevated CD206 expression and diminished CD86-related signaling align with this more refined model of ATM biology.

The reduction in adiposity and improved perivascular adipose tissue function due to exercise contributes to overall better cardiovascular health, including protection against hypertension in obese mice [31,32]. Here, we first examined the effects of exercise on the VAT weight in a hypertensive mouse model. To reveal the relationship between body weight and VAT weights, we normalized the VAT weights to the body weights. As previously shown that exercise training significantly reduces VAT weight (fat mass) in obese mice [33,34], our results also demonstrated that there was a significant decrease in the ratio of VAT/body weight of the hypertensive mice in the exercised group when compared to the sedentary group. Moreover, aerobic exercise could improve the adipose tissue function by modulating the protein and lipid composition of adipose tissue HFD-induced adiposity [35]. Therefore, we looked into the protein concentration in the VAT. Results showed that the expression of the protein was decreased in the exercise hypertensive group compared to the no-exercise hypertensive group. All that data indicate that the exercise training could affect the VAT in the hypertensive mice.

In addition to assessing histological change, we investigated inflammation-related mechanisms within AT. The AT in a healthy individual maintains vascular homeostasis through endocrine and paracrine pathways. However, an increase in VAT depots may lead to an imbalance in the levels of adipokines and can lead to inflammation of VAT during hypertension [36]. In the present study, we measured the macrophage polarization in VAT by staining the M1/M2 macrophages. As shown in the previous studies, the exercise-induced adaptations to AT involve altered mitochondrial activity [18,37], decreased cell size and lipid content [38], and reduced inflammation [39,40] in rodents. The data proved our original hypothesis that there was an increase in pro-inflammatory macrophages (M1) staining in the VAT of hypertension, and exercise attenuated the intensity of M1. Unexpectedly, a significant decrease in the anti-inflammatory macrophages (M2) was observed in the hypertensive mice. However, the exercise had a significant effect on increasing the M2 intensity in the VAT of hypertensive mice.

To further elucidate the underlying mechanisms of the inflammation, we performed flow cytometry to determine the numbers of macrophages and define the population of M1/M2 macrophages in the VAT. As anticipated, the population of M1 macrophages was increased in the VAT of hypertensive mice. More importantly, exercise decreased the M1 population in the VAT of hypertensive mice. Similar to the histology data, no significance was observed in the decrease in M2 macrophage population in the VAT of hypertensive mice w/wo exercise. Accordingly, the ratio of M1/M2 was significantly increased in the VAT of hypertensive mice. More importantly, exercise could significantly decrease the M1/M2 ratio in the VAT of hypertensive mice. Based on these results, exercise could provide beneficial effects on hypertension-induced inflammation by modulating the polarization of macrophages in the VAT. The variation in results between flow cytometry and Western blot illustrates the unique biological metrics obtained from each method. Flow cytometry determines the proportion of CD206^+^ macrophages in the stromal vascular fraction, whereas Western blot assesses the overall CD206 protein in the entirety of VAT. Exercise could elevate CD206 expression per cell while not significantly affecting the proportion of CD206^+^ macrophages, leading to a notable rise in protein levels but an insignificant change in cell percentages. Moreover, the tissue digestion processes necessary for flow cytometry led to inconsistencies in marker detection, while Western blotting offers greater sensitivity for overall protein-level variations.

As we observed that the protein concentration was increased in the VAT, the Western blot was performed to reveal specific proteins by targeting the macrophage phenotypes (polarization). Consistent with histology and flow cytometry data, the protein blotting data showed that the M1 protein was increased in the VAT of hypertensive mice, suggesting pro-inflammatory activity in the VAT of hypertensive mice. In contrast, the M2 protein was decreased in the hypertensive mice, confirming the pro-inflammatory state in the VAT of hypertensive mice. Of note, the exercise provided the beneficial effects on alleviating the inflammatory status in VAT by promoting the anti-inflammatory macrophage and compromising the pro-inflammatory macrophages.

Collectively, these results endorse a framework where exercise alleviates hypertension-related VAT impairment by diminishing adipocyte enlargement, lowering ECM buildup, and altering macrophage activation towards tissue-repair and anti-inflammatory characteristics. These modifications enhance the noted advancements in vascular remodeling and systemic inflammation associated with exercise. By contextualizing these findings within a contemporary perspective on ATM variability, our research enhances the increasing recognition of adipose–immune–vascular interactions in hypertension.

## 4. Materials and Methods

### 4.1. Animals

Renin transgenic (R+) hypertensive mice and their matched control littermates were bred in the Laboratory Animal Resource (LAR) facility. Animals were housed in standard cages at 22 °C under a 12-h light/12-h dark cycle with ad libitum access to water and standard mouse chow. All the animal experimental procedures (protocol 763) were approved by the Wright State University laboratory and the Marshall University Laboratory Animal Care and Use Committee (LACUC) and followed the Guide for the Care and Use of Laboratory Animals issued by the National Institutes of Health.

### 4.2. Exercise Procedure and Group Information

Hypertensive and control mice (5–7 weeks old, male to female: 50% to 50%) were randomly divided into 3 different groups: (1) control mice without exercise (control group), (2) renin transgenic mice without exercise (R^+^ + no exercise), and (3) renin transgenic mice with exercise (R^+^ + exercise). Exercised mice were subjected to exercise training for one week on a treadmill (Columbus Instruments) for one hour per day, five days a week per our previous publication [41,42]. The Initial speed was set at 6.0 m/min and gradually increased by 1 m/min until 10 m/min by the end of the training. In the subsequent exercise group, mice were run at 10 m/min, one hour per day, five days a week, for 8 weeks. All mice were monitored weekly for body weight. At the end of the experiment, aorta, blood, and visceral adipose tissue samples were collected for different analyses.

### 4.3. Blood Pressure Recording

A radiotelemetry system (TA11PA-C10, Data Science International) was used for recording arterial blood pressure (BP) under anesthesia by inhaling 2.5% isoflurane as we reported previously [43]. The telemetric probes were implanted in the left carotid artery. BP was recorded for 24 h before exercise and right after exercise (week 8). We recorded BP for 10 min (sample rate 500 Hz) once an hour for 24 h to calculate the mean arterial pressure (MAP) for each day. The MAP was started to be recorded at the same time for each mouse.

### 4.4. Samples Collection

After 8 weeks of exercise, mice were euthanized immediately by deep anesthetization with an i.p. injection of pentobarbital (150 mg/kg). Once the mice were under deep anesthesia, a midline incision was made in the thoracic cavity to expose the abdomen. Blood was collected from the heart of mice using a 1 mL syringe containing a small amount of 0.5 M EDTA for anticoagulation. Blood samples were centrifuged at 2000× *g* for 10–15 min. Plasma was immediately separated and stored frozen at −80 °C for future use. Cardiac perfusion was performed after blood sample collection by using 1× phosphate-buffered saline (PBS) to remove the rest of the blood, followed by 4% paraformaldehyde (Thermo Fisher Scientific, Waltham, MA, USA) to pre-fix the samples. The aorta and VAT were collected and weighed. The tissue was rinsed with PBS to remove any contaminants, such as fur, and placed in a lysis buffer for protein extraction or formalin for the histology process.

### 4.5. H&E Staining

Aorta and VAT were fixed in 10% formalin overnight and embedded in paraffin the next day. Tissue samples were cut into 10 μm sections and mounted on glass slides. Slides were dewaxed in xylene and rehydrated in graded alcohols. The slides were then stained in hematoxylin solution (Fisher Scientific, Waltham, MA, USA) for 15 min and washed in double-distilled water for 1 min. After rinsing in water, the slide was placed in Scott’s tap water substitute (1:10) for 5 s. A subsequent rinse was performed in water and then stained with an eosin Y solution (Fisher Scientific, Waltham, MA, USA) for 1–2 min. The slide was rinsed in xylene again and subjected to different grades of alcohol to rehydrate. Images were taken on a confocal laser scanning microscope (Olympus, Center Valley, PA, USA). Image J software 1 (NIH) was used to measure the remodeling of the aorta (wall/lumen ratio), the cell nuclei numbers, and the cell area of adipocytes.

### 4.6. Masson Trichrome Staining

The slides contain VAT were transferred to Bouin’s fluid (Abcam, Waltham, MA, USA) for 1 h, and rinsed in tap water followed by distilled water. The slides were then stained with a working Weigert’s hematoxylin solution (Abcam) for 5 min and rinsed in running tap water for 2 min. A Biebrich Scarlet/Acid Fuschin Solution (Abcam) was applied to the slides for 15 min, followed by a rinse in distilled water. The slides were then differentiated in Phosphomolybdic/Phosphotungstic Acid Solution (Abcam) for 15 min, and followed immediately by application of Aniline Blue Solution (Abcam) for 10 min and a rinse in distilled water. The 1% Acetic Acid Solution (Abcam) was then applied to the slides for 5 min. Images were taken on a confocal laser scanning microscope (Olympus) under 10× and 20× objectives. Image J software (NIH) was used to measure the collagen frequency in the adipose samples in relation to the image area.

### 4.7. Immunohistochemistry (IHC) Staining

Immunohistochemistry DAB staining was used to detect the polarization of macrophages (M1/M2) in VAT with specific biomarkers. VAT was processed as described in the H&E staining section. After the slides were dewaxed and rehydrated, the slides were transferred to a sodium citrate solution for antigen retrieval for 1 h at 60 °C. After the antigen retrieval was completed, the slides were given approximately 5 min to cool before adding them to any other reagents. After the cooling was complete, the slides were washed in PBS Triton X-100 solution (Fisher Scientific, Waltham, MA, USA) 3 times for 5 min each. After this, the slides were treated with blocking buffer (1% BSA in PBS-T) for 2 h. Then, slides were incubated in the appropriate primary antibody (CD86 for M1, CD206 for M2, 1:500, Abcam) overnight at 4 °C. When slides were obtained the next day, the primary antibody was removed, and the slides were again washed in PBS-T for three 5-min washes. The slides were then treated with 0.3% H_2_O_2_ diluted in PBS-T for 15 min, followed by another three consecutive PBS-T washes and treatment for 1 h with a dilution of the secondary antibody (HRP anti-mouse, 1:500, BioRad, Hercules, CA, USA) in blocking buffer for 1 h. After the secondary antibody treatment and another cycle of washes, the slides were treated with DAB chromogen for 10 mins and washed again. After this, the slides were counterstained for 2.5 min in hematoxylin. These slides were then dehydrated using increasing concentrations of alcohols and xylenes. Image J software (NIH) is used to measure the frequency of macrophage species within a certain image area.

### 4.8. Flow Cytometry

VAT was minced using a digestion buffer containing 2 mg/mL of collagenase in Hanks balanced salt solution with Ca^2+^ and Mg^2+^ supplemented with 0.5% bovine serum albumin (BSA, Sigma-Aldrich). The tissue was then incubated at 37 °C for 90 min with vigorous shaking every 5 min. After digestion, 10 mM EDTA was added and incubated at 37 °C for an additional 5–10 min. A 100 μm nylon filter was prewet with PBS and placed on a conical tube. The bottom layer of the slurry was transferred onto the filter, followed by the adipocyte-containing upper layer. Then the cell slurry was centrifuged at 500× *g* for 10 min at 4 °C to separate adipocytes from the stromal vascular cells. After centrifugation, the adipocytes form a white layer, and the stromal vascular cells form a red/white pellet on the bottom of the tube. Adipocytes and supernatant were gently aspirated and discarded. The pellet was disrupted by flicking the tube and resuspended in 0.5 mL red blood cells (RBC) lysis buffer and incubated at room temperature with occasional shaking. RBC lysis buffer was prepared with 155 mM NH_4_CL, 10 mM KHCO_3_, 0.1 M EDTA; sterile-filtered through 0.22 μm filter, and stored in aliquots at 4 °C. RBC lysis buffer was neutralized by adding 5 mL of FACS buffer, which was prepared with PBS with 1 mM EDTA, 25 mM HEPES, and 1% heat-inactivated fetal bovine serum. The solution was centrifuged at 500× *g* for 10 min at 4 °C in MACS buffer (PBS without Ca^2+^ and Mg^2+^ supplemented with 0.5% BSA). Cells were suspended in 90 μL of PBS and incubated with F4/80-FITC (20 μL, BD Sciences), CD206-APC (20 μL, BD Biosciences, Bedford, MA, USA), and CD86-PE (10 μL, BD Biosciences, Bedford, MA, USA). Samples were analyzed by a flow cytometer and C flow plus analysis software (Accuri C6 flow cytometer, BD Biosciences, Bedford, MA, USA).

### 4.9. Western Blot Analysis

Proteins from VAT were obtained with lysis buffer (Thermo Scientific, FL, USA) containing protease inhibitors. The extracted proteins were analysed by Bicinchoninic Acid (BCA) colorimetric assay. The proteins were subjected to electrophoresis and transferred onto nitrocellulose membranes. The membranes were blocked by incubating with 5% dry milk for 1 h, and then incubated with primary antibodies: against CD206 (1:1000; Abcam, Waltham, MA, USA), CD86 (1:1000; Abcam, Waltham, MA, USA), at 4 °C overnight. After being washed thoroughly, membranes were incubated with horseradish peroxidase (HRP) conjugated IgG (1:40,000; Jackson ImmunoResearch Labs, INC. West Grove, PA, USA) for 1 h at RT. β-actin (1:4000; Sigma-Aldrich, St. Louis, MO, USA) was used to normalize protein loading.

### 4.10. Statistical Analysis

Data are represented as mean ± SEM. Multiple comparisons among the groups were carried out by two-way ANOVA followed by post hoc Tukey’s test. A value of *p* < 0.05 was considered significant. All statistical analyses were performed using GraphPad Prism software version 10.0 (GraphPad Software, La Jolla, CA, USA). All analyses were conducted blind to the experimental conditions.

## 5. Conclusions

In summary, this is the first study to demonstrate that exercise has beneficial effects on vascular remodeling by modulating the inflammatory status in the visceral adipose tissue in a hypertensive mouse model. Future studies are needed to look into how exercise and metabolic stimuli modulate macrophage phenotype changes in different subsets, such as the resident, perivascular, lipid-associated, and infiltrating populations. Moreover, more animals are needed to study the sex- and age-dependent effects of exercise.

## Data Availability

The original contributions presented in this study are included in the article. Further inquiries can be directed to the corresponding author.
